# Development and validation of a PMA-qPCR method for accurate quantification of viable *Lacticaseibacillus paracasei* in probiotics

**DOI:** 10.3389/fmicb.2024.1456274

**Published:** 2024-08-07

**Authors:** Lizheng Guo, Xiaolei Ze, Yingxin Jiao, Chengyu Song, Xi Zhao, Zhiquan Song, Shuaicheng Mu, Yiru Liu, Yuanyuan Ge, Yu Jing, Su Yao

**Affiliations:** ^1^China Center of Industrial Culture Collection, China National Research Institute of Food and Fermentation Industries Co., LTD., Beijing, China; ^2^Microbiome Research and Application Center, BYHEALTH Institute of Nutrition & Health, Guangzhou, China

**Keywords:** probiotics, *Lacticaseibacillus paracasei*, PMA-qPCR, identification, viable cell quantification, method validation

## Abstract

The effectiveness of probiotic products hinges on the viability and precise quantification of probiotic strains. This study addresses this crucial requirement by developing and validating a precise propidium monoazide combination with quantitative polymerase chain reaction (PMA-qPCR) method for quantifying viable *Lacticaseibacillus paracasei* in probiotic formulations. Initially, species-specific primers were meticulously designed based on core genes from the whole-genome sequence (WGS) of *L. paracasei*, and they underwent rigorous validation against 462 WGSs, 25 target strains, and 37 non-target strains across various taxonomic levels, ensuring extensive inclusivity and exclusivity. Subsequently, optimal PMA treatment conditions were established using 25 different *L. paracasei* strains to effectively inhibit dead cell DNA amplification while preserving viable cells. The developed method exhibited a robust linear relationship (*R*^2^ = 0.994) between cycle threshold (C_q_) values and viable cell numbers ranging from 10^3^ to 10^8^ CFU/mL, with an impressive amplification efficiency of 104.48% and a quantification limit of 7.30 × 10^3^ CFU/mL. Accuracy assessments revealed biases within ±0.5 Log_10_ units, while Bland–Altman analysis demonstrated a mean bias of 0.058 Log_10_, with 95% confidence limits of −0.366 to 0.482 Log_10_. Furthermore, statistical analysis (*p* = 0.76) indicated no significant differences between theoretical and measured values. This validated PMA-qPCR method serves as a robust and accurate tool for quantifying viable *L. paracasei* in various sample matrices, including pure cultures, probiotics as food ingredients, and composite probiotic products, thereby enhancing probiotic product quality assurance and contributing to consumer safety and regulatory compliance.

## 1 Introduction

Probiotics, live microorganisms beneficial to human health when consumed in appropriate quantities, offer diverse advantages such as alleviating lactose intolerance, reducing obesity, and enhancing gut microflora (The World Health Organization, 2001; Principi et al., 2018; Son et al., 2018; Jang et al., 2019; Song et al., 2023). Their extensive utility spans various sectors including food, cosmetics, dietary supplements, and pharmaceuticals, underscoring their importance in promoting human well-being (Kumar et al., 2015; Quin et al., 2018; Song et al., 2023). However, the efficacy of probiotics relies heavily on the specific strains used and their viability, which are influenced by factors like manufacture method, fermentation processes, and storage conditions (Fenster et al., 2019; Beck et al., 2022; Congjie et al., 2024; Hellebois et al., 2024; Wang and Zhong, 2024). Therefore, accurate quantification of viable cells, particularly in compound probiotic products, is critical for ensuring product quality, regulatory compliance, and consumer safety. This quantification not only verifies promised health benefits but also fosters market competitiveness and drives scientific innovation within the probiotic industry.

**Figure 1:**
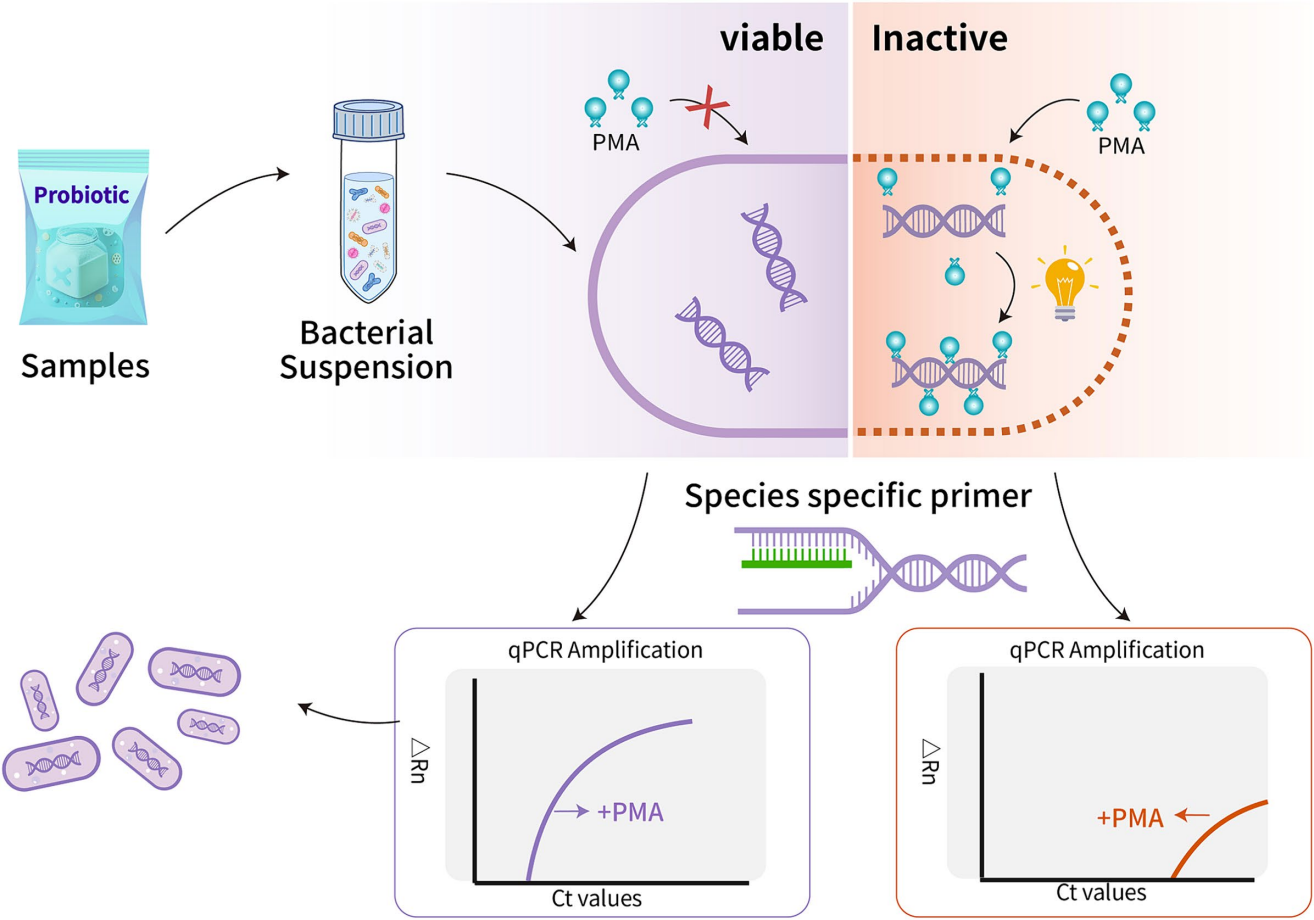
It illustrates the principle of viable cell counting using the PMA-qPCR method. This technique relies on cell membrane integrity and the use of specific primers to selectively enumerate viable cells of targeted probiotics in compound products. Viable cells with intact membranes are distinguished from non-viable cells, allowing for accurate quantification of viable probiotics present.

Currently, culture-based methodologies face inherent challenges in differentiating or selectively enumerating probiotics in compound products, failing to meet the demands of the probiotic industry (Boyte et al., 2023; Sibanda et al., 2024). Nucleic acid-based methods, particularly quantitative PCR (qPCR), have gained widespread acceptance across various disciplines such as biology, food science, and environmental science due to their rapidity, specificity, and exceptional sensitivity (Guo et al., 2020; Boyte et al., 2023; Shehata et al., 2023). When combined with propidium monoazide (PMA) dye, PMA-qPCR facilitates the quantification of viable cells through selective staining based on membrane integrity (Nocker et al., 2006; Guo et al., 2024; Marole et al., 2024). The PMA dye selectively penetrates membrane-damaged cells, forming covalent cross-links with DNA upon photolysis, preventing subsequent PCR amplification of DNA from dead cells. Consequently, DNA from membrane-intact cells is selectively amplified in the subsequent PCR procedure (Figure 1) (Nocker et al., 2006; Scariot et al., 2018; Shehata et al., 2023).

When applying PMA-qPCR, it is essential to consider several crucial factors that would impact the results, depending on the target strains and sample types. Firstly, the design of specific primers is fundamental in qPCR, as it ensures detection accuracy, enhances sensitivity, and minimizes false positives and negatives (Kwon et al., 2005; Fujimoto and Watanabe, 2013; Zhao et al., 2022; Kiousi et al., 2023). The efficiency of DNA extraction is another critical factor, as it directly affects how accurately the qPCR results reflect the biomass in the samples. Therefore, selecting an appropriate DNA extraction method based on the sample type is vital for obtaining accurate and stable results (Douglas et al., 2020; Shetty and Mariyam, 2020). Additionally, the PMA treatment conditions must be optimized to fully inhibit the amplification of DNA from dead cells without significantly affecting the detection of viable cells, which is crucial for accurate viable cell counts (Zhang et al., 2020; Latka et al., 2022). Another important factor is the qPCR amplification program, which impacts the amplification efficiency of the primers and affects the standard curve. This, in turn, influences the relationship between the C_q_ values and gene copies or viable cell numbers. Finally, the selection of strains used to construct the standard curve and its applicability to different strains within a species should be carefully considered (Svec et al., 2015; Ilha et al., 2016; Odooli et al., 2018; Scariot et al., 2018; Ruijter et al., 2021).

The performance parameters of microbiological methodologies are recommended to be evaluated and validated to ensure that they are suitable for their intended use. Quantitative techniques like PMA-qPCR demand meticulous assessment of accuracy, precision, specificity, quantification limit, linearity, and ruggedness (Broeders et al., 2014). These metrics are crucial for determining the method’s robustness and reliability across diverse applications. The PMA-qPCR method, renowned for its ability to differentiate between live and dead cells based on membrane integrity, holds significant promise for accurately quantifying viable cells, especially in complex matrices like compound probiotic products. However, despite its widespread application in various sectors, comprehensive evaluation of its efficacy in quantifying specific target species is often lacking. Thorough validation of the PMA-qPCR method is essential to ensure its precision and reliability across different applications, supporting scientific research, quality control, and regulatory compliance. Through method validation, the effectiveness of PMA-qPCR can be improved in real-world scenarios, ensuring that probiotic products meet their intended health benefits and maintain high standards of quality and safety. This, in turn, enhances public health and fosters consumer trust in these products.

*Lacticaseibacillus paracasei*, recognized as a pivotal probiotic resource, assumes a prominent role within the global health food industry. At present, several commercially available strains of *L. paracasei* find widespread application in the production of dairy items, solid beverages, and health supplements (Zhang et al., 2010; Falfán-Cortés et al., 2022; Pérez Martínez et al., 2023; Beverage et al., 2024). Furthermore, *L. paracasei* manifests commendable physiological effects, exerting a pivotal role in modulating the equilibrium of the human intestinal microbiota (Chuang et al., 2011) and serving as a probiotic in disease prevention (Chiang and Pan, 2012). Notably, it demonstrates the capability of maintaining stable viability within the human intestinal tract, positioning it as a promising candidate for incorporation into functional foods. Particularly, the domain of dairy product development stands out as an area with substantial potential for future advancement. In this study, a precise PMA-qPCR method for quantifying viable *L. paracasei* was developed and rigorously evaluated. A species-specific primer pair was meticulously designed based on core genes identified in the whole genome sequence of *L. paracasei*. The validation process for these primers encompassed comprehensive inclusivity and exclusivity testing, conducted through whole-genome sequence blasts and a thorough analysis of strains collected at various taxonomic levels. The efficacy of the PMA treatment conditions was verified using 25 different *L. paracasei* strains, ensuring that the method did not interfere with the PCR amplification of viable cells while effectively suppressing the amplification of non-viable cells. A standard curve correlating qPCR C_q_ values with viable bacterial counts was constructed. The PMA-qPCR method was then applied to a variety of samples, demonstrating its relative trueness, accuracy, linearity, limit, and quantification range. This study successfully established a robust PMA-qPCR method for accurately quantifying viable *L. paracasei* in heterogeneous samples, offering valuable implications for evaluating the viability and quality of probiotic products.

## 2 Materials and methods

### 2.1 Specific primer design

After executing data quality control and conducting an analysis of average nucleotide identity (ANI), we acquired 176 publicly available genomes of *L. paracasei* from the National Center for Biotechnology Information (NCBI). These genomes underwent re-annotation utilizing Prokka v1.14.6 to identify protein sequences, and the format was standardized to align with that of the 15 self-sequenced genomes. Subsequently, a gene presence/absence analysis was conducted based on the annotated protein sequences. Gene families of *L. paracasei* were individually constructed using the CD-HIT rapid clustering of similar proteins software (v4.6), applying a threshold value of 50% pairwise identity and a 0.7 length difference cutoff in amino acids (Li and Godzik, 2006; Li et al., 2008). The genes only present in all the infraspecific strains were preliminarily identified as the core genes in *L. paracasei*. In consideration of the gene presence/absence analysis being carried out at the protein level, the nucleotide specificity of these conserved genes was subsequently verified through BLASTN against the NCBI Nucleotide collection (NT) (Altschul et al., 1990). The species-specific gene for Alkaline shock protein 23 was identified for primer design. Subsequently, the corresponding PCR primer pairs for *L. paracasei* were meticulously crafted using Primer Premier v6.0, with adherence to various design principles (Singh et al., 1998; Elsalam, 2003). The primer, designed with a length of 180 bp (Lpa-F: 5’-ACGCTGGCATCAATAAGGAATT-3′; Lpa-R: 5’-CATCGCTCAGGTCTACATCCA-3′), was synthesized by Sangon Biotech (Shanghai, China).

### 2.2 Inclusivity and exclusivity validation of primer specificity

Inclusivity was conducted to assess primer ability to detect target strains (ISO 16140-2, 2016). Firstly, the primer was assessed *in silico* through aligning with 38 whole-genome sequences (WGS) of *L. paracasei* through Primer-BLAST on NCBI^1^ (Ye et al., 2012; Lawley et al., 2017; Guo et al., 2024). The WGS sequences were downloaded from NCBI website^2^ including type strain, commercial strains, and others. Then, the primer was further validated by PCR test using the DNA templates extracted from 25 different *L. paracasei* strains (Table 1). The thermal cycling of PCR assay consisted of initial denaturation at 95°C for 5 min, followed by 35 cycles of 95°C for 30 s, 60°C for 34 s and 72°C for 25 s, followed by a final extension step of 72°C for 10 min. The electrophoresis on 1% agarose gel was used to examine the amplification products using Gel Doc EZ System (Bio-Rad, California, USA).

**Table 1:** Bacterial strains used in this study.

Inclusivity and exclusivity study	Genus	Strains
Inclusivity	*Lacticaseibacillus*	*L. paracasei* CICC 6263^T^, 6264^T^, CICC 6028, CICC 6110, CICC 6138, CICC 6227, CICC 20241, CICC 20266, CICC 22165, CICC 22829, CICC 22830, CICC 22709, CICC 24700, CICC 24825, Z-022, 8130T, ET-22, K56, LC01, Shirota, LPC-37, 431, LC-37, Zhang, 207–27
Exclusivity	*Lacticaseibacillus*	*L. casei* CICC 6117^T^, *L. rhamnosus* CICC 6224^T^, *L. zeae* CGMCC 1.2442
	*Bifidobacterium*	*B. animalis* subsp. *lactis* CICC 24210^T^, *B. animalis* subsp. *animalis* CICC 6250^T^, *B. adolescentis* CICC 6070^T^, *B. breve* CICC 6079^T^, *B. longum* subsp. *longum* CICC 6186^T^, *B. longum* subsp. *infantis* CICC 6069^T^, *B. bifidum* CICC 6071^T^
	*Lactobacillus*	*L. acidophilus* CICC 6081^T^, *L. crispatus* JCM 1185^T^, *L. delbrueckii* subsp. *bulgaricus* CICC 6103^T^, *L. delbrueckii* subsp. *lactis* CGMCC 1.2625^T^, *L. gasseri* CICC 24878^T^, *L. helveticus* CICC 24208^T^, *L. johnsonii* CICC 6252^T^, *L. kefiranofaciens* subsp. *kefiranofaciens* CGMCC 1.3402^T^
	*Limosilactobacillus*	*L. fermentum* CICC 24209^T^, *L. reuteri* CICC 6132^T^
	*Lactiplantibacillus*	*L. plantarum* CICC 6240^T^
	*Ligilactobacillus*	*L. salivarius* CGMCC 1.1881^T^
	*Latilactobacillus*	*L. curvatus* JCM 1096^T^, *L. sakei* CICC 6245^T^
	*Streptococcus*	*S. salivarius* subsp. *thermophilus* CICC 6222^T^
	*Lactococcus*	*L. lactis* subsp. *lactis* CICC 6246^T^, *L. cremoris* CICC 24337^T^
	*Propionibacterium*	*P. freudenreichii* subsp. *shermanii* CGMCC 1.2231^T^, *P. acidipropionici* CICC 24923^T^
	*Leuconostoc*	*L.* subsp. *mesenteroides* CICC 25070^T^, *L. mesenteroides* subsp. *cremoris* CICC 22181
	*Pediococcus*	*P. acidilactici* CGMCC 1.2696^T^, *P. pentosaceus* CGMCC 1.2695^T^
	*Weizmannia*	*W. coagulans* CGMCC 1.2009^T^
	*Staphylococcus*	*S. vitulinus* CICC 10850, *S. xylosus* JCM 2418^T^, *S. carnosus* ACCC 01657

The exclusivity of primer characterizes the non-detection of non-target strains (ISO 16140-2, 2016). Seventy whole-genome sequences (WGS) of 25 strains of *Lacticaseibacillus* at the species level, 281 WGS of 30 strains within the family *Lactobacillaceae* at the genus level, and 73 WGS of the 36 strains listed in the Chinese catalog of food-safe cultures were downloaded from the NCBI. The primer was assessed *in silico* by aligning with these WGS through Primer-BLAST on NCBI (Ye et al., 2012; Lawley et al., 2017). Then, 36 strains in Chinese list of cultures that can be used for food and *L. zeae* were collected (Table 1). The DNA templates of these strains were isolated and PCR products were imaged by 1% agarose gel electrophoresis under UV lights.

### 2.3 Genomic DNA extraction

The bead-beating methods were demonstrated effectiveness for DNA extraction (Fujimoto et al., 2004; Guo et al., 2024). Briefly, the screw-cap 2.0 mL sample tubes containing 0.25 g of Zirconia/Silica beads with 0.1 mm were autoclaved. Bacterial suspensions within 200 μL of ddH_2_O were aspirated into these tubes. The BEAD RUPTOR 12 (OMNI International, USA) served as the mechanical cell disruptor for 12 s at a speed setting of 6.0 m/s. Supernatants containing DNA were obtained by centrifuged at 12,000 rpm for 15 min and 50 microliters of that were transferred into 1.5 mL sterile tubes for subsequent qPCR assays.

### 2.4 Verification of optimal PMA treatment conditions

Accurate quantification of viable bacteria is closely linked to the appropriate conditions of PMA treatment. A commonly used PMA treatment condition, involving a concentration of 50 μM/L, followed by 5 min of dark incubation, and finally, 15 min of exposure to light, was selected (Desfossés-Foucault et al., 2012; Villarreal et al., 2013; Guo et al., 2024). To validate the suitability of the chosen PMA conditions across various *L. paracasei* strains, we deliberately selected 25 distinct strains (Table 1) for further investigation. Firstly, the bacteria were initially resuscitated on MRS solid medium at 37°C for 48 h. Subsequently, the cells were inoculated onto MRS solid medium and cultured for another 48 h. Having undergone dual cultivation on MRS solid medium under optimal conditions, the majority of the bacteria were considered highly active. The resuspended bacteria within 0.85% sodium chloride solution were adjusted to OD_620_ = 0.3–0.5 with approximate 10^8^ CFU/mL, which was further validated by plating counts. Each strain was categorized into live and dead groups. To obtain the dead groups, the bacteria were subjected to heating at 90°C for 10 min. Subsequently, both live and dead bacterial suspensions, each containing approximately 10^8^ CFU/mL, were divided into PMA treatment and non-treatment groups. The PMA solution from BIORIGIN (China) was dissolved in ddH_2_O to create a 20 mmol/L stock solution. Subsequently, 1.25 μL of this stock solution was added to 500 μL of cell suspensions, resulting in a final PMA concentration of 50 μM. The mixed samples were then placed in the dark for 5 min to allow PMA to penetrate dead cells and bind to their DNA. Following this incubation, the treated samples were exposed to a 60 W LED light source (Biotium, USA) for 15 min. Subsequently, both the bacterial suspensions from the PMA treatment group and the non-treatment group were centrifuged at 12,000 rpm for 15 min. The harvested bacterial pellets were then subjected to DNA extraction.

### 2.5 Quantitative PCR amplification

The total qPCR volume was 20 μL per reaction, including 10.0 μL of 2 × SYBR Green premix (TaKaRa, Japan), 0.4 μLof each10μM forward and reverse primers, 0.08 μL of ROX reference dye, 2 μL of bacteria genomic DNA, and 7.12 μL ddH_2_O. The thermal cycle program was as follows: 95°C for 30 s, followed by 40 cycles of 95°C for 5 s, 60°C for 34 s. The qPCR reactions were carried out in an ABI 7500 Fast real-time PCR system. Triplicates were performed for target DNA and sterile water (negative control).

### 2.6 Construction of standard curves between viable cell numbers and C_q_ values

In order to achieve viable cell counting by PMA-qPCR method, a standard curve between viable cell numbers and qPCR C_q_ values was performed (Ilha et al., 2016; Odooli et al., 2018; Scariot et al., 2018). Fresh cultures of *L. paracasei* CICC 6263^T^ was obtained and then diluted to 10^8^ CFU/mL that further confirmed by culture plating. The bacteria with 10^8^ CFU/mL were treated by PMA to filter dead cells and then DNA was extracted as described above. The DNA series with 10-fold dilutions was amplified to obtain the C_q_ values. Then, the standard curve between C_q_ values and viable cell numbers were constructed.

### 2.7 Linear and quantification limits of the PMA-qPCR method

Samples were prepared by combining viable *L. paracasei* cells with nonviable cells of *L. rhamnosus*. In each sample, a consistent count of nonviable *L. rhamnosus* cells was maintained at approximately 10^8^ CFU/mL, while varying concentrations of viable *L. paracasei* cells were introduced, namely 10^8^, 10^7^, 10^6^, 10^5^, 10^4^, and 10^3^ CFU/mL. The 10^3^ CFU/mL were further diluted to obtain lower *L. paracasei* concentration for quantification limits detection. Viable *L. paracasei* were quantified using the culture-based method to obtain the theoretical values. To obtain the linear characteristics, the PMA-qPCR measured values and theoretical values were linearly fitted.

### 2.8 Quantification of viable *Lacticaseibacillus paracasei* by PMA-qPCR method

The wide applicability of the established PMA-qPCR method was firstly confirmed using 25 strains of *L. paracasei* (Table 1). These strains included type strains CICC 6263^T^, CICC 6264^T^, and commercial strains Z-022, 8130T, ET-22, K56, LC01, Shirota, LPC-37, 431, LC-37, Zhang, 207–27, as well as CICC 6110, CICC 22165, CICC 20241, CICC 22830, CICC 6138, CICC 6028, CICC 24700, CICC 24825, CICC 6227, CICC 20266, CICC 22709, and CICC 22829. Each bacterial suspension was adjusted to approximately 10^8^ CFU/mL, followed by PMA treatment, DNA extraction, and qPCR amplification. Viable cell number of each strain were further determined by plate counting.

As probiotics used in food ingredients typically contain high concentrations of bacteria, a selection of six probiotic formulations comprising singular *L. paracasei* strains (e.g., zhang, LPB-27, etc.) or combinations with other probiotics and lactic acid bacteria, along with simple excipients such as maltodextrin, were collected. Initially, the total bacteria of each sample were diluted to approximately 10^8^ CFU/mL, and viable numbers of *L. paracasei* were detected using the established PMA-qPCR method. Theoretical values of *L. paracasei* in each sample were provided by the producer.

To further validate the PMA-qPCR method’s capacity to accurately quantify viable *L. paracasei* within composite bacterial flora and withstand interference from the matrix, eight compound probiotic products were collected. Each compound probiotic product contained typically featured intricate formulations. These formulations incorporated complex excipients, including common additives such as maltodextrin and resistant dextrin, as well as prebiotics like fructooligosaccharides, erythrosis, and stachyose. Additionally, botanical ingredients such as cranberry, peach, and hawthorn powder were included in these probiotic formulations. Then, the established PMA-qPCR method was used to detect viable *L. paracasei* in these compound probiotics. Theoretical values of viable *L. paracasei* in these samples were obtained according to products claims. To enhance the analysis of probiotic products, qPCR was used on PMA-untreated samples to identify and measure dead or damaged bacteria, offering a complete view of the total bacterial count, encompassing both living and dead cells. The principles of how PMA-qPCR quantifies the number of viable target cells in compound probiotics are illustrated in Figure 1.

### 2.9 Statistical analysis

The statistical analysis comprised two key methodologies. Firstly, the *T*-test method, executed in Excel (Microsoft Office 2021), was employed to determine the significance of PMA treatment conditions on viable cells, comparing treated and non-treated groups. Additionally, the *T*-test assessed the significance between theoretical and measured values across all 39 samples, with a significance threshold of *p* < 0.05. Secondly, to ensure rigorous analysis and scientific validity, the Bland–Altman method was utilized. Implemented using R software (version 4.2.2), this method evaluated the agreement between theoretical and PMA-qPCR measured results. It involved plotting individual differences against mean values, incorporating the line of identity, line of bias, and upper and lower 95% confidence limits of agreement.

## 3 Results

### 3.1 Specificity of the newly designed primer

**Figure 2:**
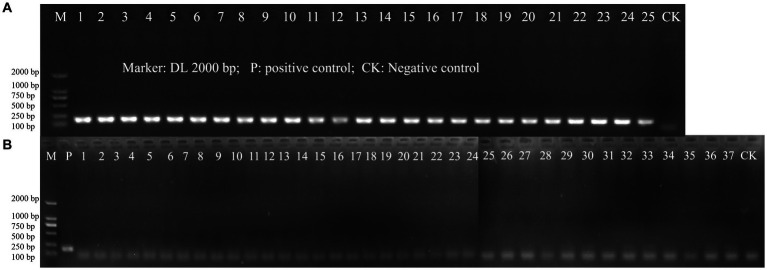
The PCR amplification of inclusivity and exclusivity assay visualized on an agarose gel. **(A)** Inclusivity assay with 25 target strains (Table 1); **(B)** Exclusivity assay with 37 non-target strains (Table 1).

The specificity of the designed primer for *L. paracasei* was initially validated through a Primer-BLAST analysis on NCBI. In this preliminary test, no significant similarity with non-target microorganisms was detected. Then, inclusivity and exclusivity of the primer were evaluated using DNA templates from 25 target strains of *L. paracasei* and 37 non-target strains by PCR amplification. Positive results for the 25 strains of *L. paracasei* were obtained, while other 37 strains were all negative (Figure 2). The results demonstrated the highly specificity of the newly designed primer to *L. paracasei* and target detection of *L. paracasei* within multi-strains would be achievable.

### 3.2 Evaluation of the optimal PMA treatment conditions

**Figure 3:**
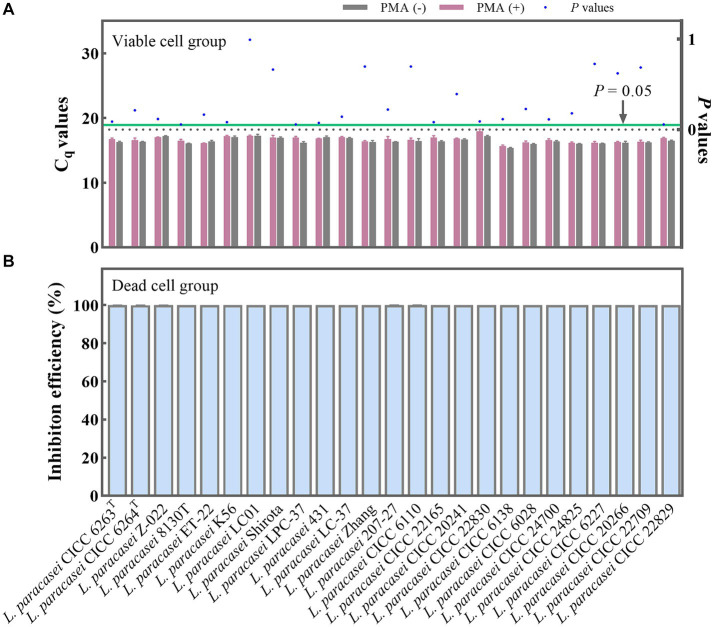
Optimal PMA treatment conditions evaluation. **(A)** Assessment of the impact of PMA treatment on qPCR amplification of viable *L. paracasei* cells from 25 different strains. PMA (+) and PMA (−) represent samples treated with and without PMA, respectively. **(B)** Determination of the inhibition efficiency of PMA on dead *L. paracasei* cells.

The PMA treatment conditions involving a concentration of 50 μM/L was selected. This was followed by 5 min of dark incubation and, finally, 15 min of exposure to light. To further validate the optimality and broad applicability of these chosen PMA treatment conditions, 25 strains of *L. paracasei* were employed with and without PMA treatment. For the viable group, the C_q_ values obtained from the PMA treatment and non-treatment groups underwent statistical analysis using the *T*-test method. No significant differences (*p* = 0.057–0.993) (Figure 3A) were observed between the treated and non-treated groups for each strain, indicating that the chosen PMA conditions would not inhibit the qPCR amplification of viable cells. The 25 different strains of *L. paracasei*, each with a concentration of 10^8^ CFU/mL, underwent heat inactivation to obtain total dead cells. Subsequently, the efficiency of PMA treatment was further evaluated. The inhibition efficiencies of PMA treatment on qPCR amplification of dead cells from each *L. paracasei* strain were calculated. As depicted in Figure 3B, the inhibition efficiency of each strain ranged from 99.96 to 100.00%. This remarkable inhibition indicates that qPCR amplification of DNA originating from dead cells was nearly completely suppressed. These results demonstrate that the chosen PMA conditions are optimal for distinguishing between viable and dead cells of *L. paracasei*, including the type strain, commercial strains, etc.

### 3.3 Conversion of C_q_ values to viable cell numbers

**Figure 4:**
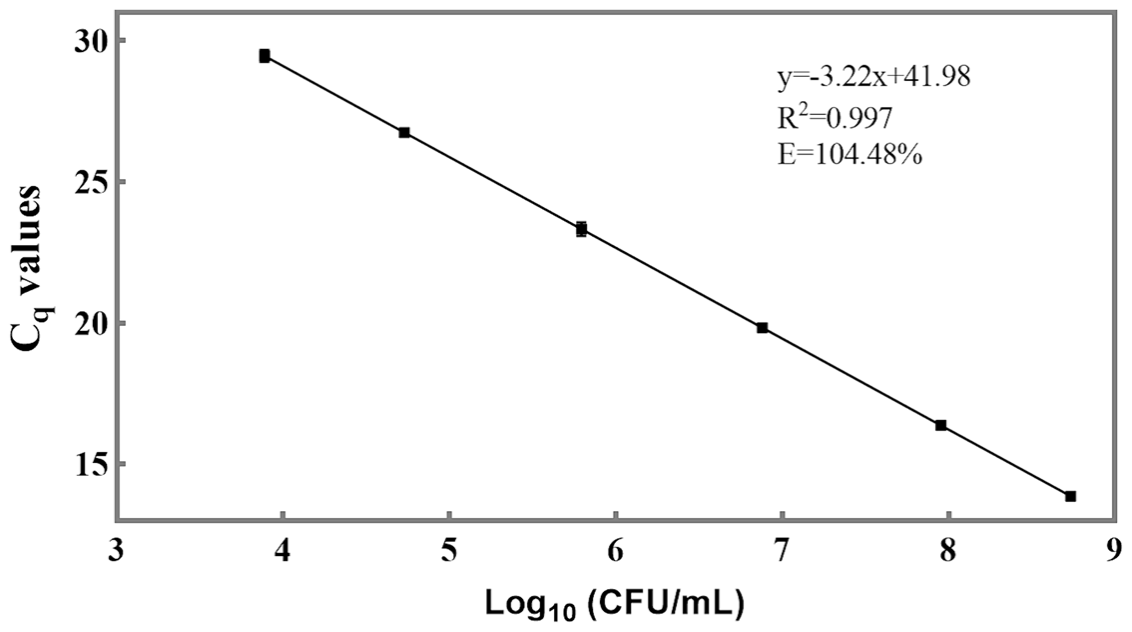
Sensitivity of the newly designed primer for quantification of viable *L. paracasei* by qPCR. The standard curve was constructed using the average C_q_ values derived from 10-fold serial dilutions of target DNA extracted from an equal proportion of *L. paracasei* and the logarithm of the concentration of culturable *L. paracasei*.

For the qPCR method, C_q_ values are the direct results obtained. To determine viable cell numbers, a relationship between C_q_ values and viable cell numbers should be established (Figure 4). The slope of the linear equation between the C_q_ values of individual strains and the logarithm of the number of viable bacteria is −3.22, and R^2^ is 0.997. The amplification efficiency (*E*) was calculated as 104.48% using the formula *E* = 10 ^(−1/slope)^ – 1 (Rogers-Broadway and Karteris, 2015; Svec et al., 2015). This efficiency value is deemed acceptable as it falls within the range of 90 to 110% (Ruijter et al., 2009), indicating that the newly designed primer also exhibits good sensitivity and can be utilized for the detection of *L. paracasei* in probiotic products. Through the utilization of the standard curve, it became feasible to convert the C_q_ values of *L. paracasei* samples into CFU equivalent cells.

### 3.4 Limit of quantification of the established PMA-qPCR method

To ascertain the limit of quantification of the established PMA-qPCR method, three composite samples were prepared, each containing viable *L. paracasei* and non-viable *L. rhamnosus*. In each sample, the concentration of non-viable *L. rhamnosus* remained approximately 10^8^ CFU/mL, while viable *L. paracasei* concentrations were 2.57 × 10^3^ CFU/mL, 7.30 × 10^3^ CFU/mL, and 1.54 × 10^4^ CFU/mL, respectively. Five replicates were run for each sample. The average C_q_ values corresponding to 7.30 × 10^3^ CFU/mL and 1.54 × 10^4^ CFU/mL were 29.54 ± 0.21 and 28.49 ± 0.04, respectively, both falling below 30 and within the range of the standard curve (Figure 4). When the concentration of *L. paracasei* were 2.57 × 10^3^ CFU/mL, the average C_q_ value was 31.01 ± 0.23, which was close to the negative control and beyond the range of the standard curve. Therefore, the limit of quantification for the PMA-qPCR method was established as 7.30 × 10^3^ CFU/mL.

### 3.5 Linear, and range of the established PMA-qPCR method

In this study, compound samples comprising viable *L. paracasei* and deceased *L. rhamnosus* were prepared, with total bacterial concentrations of approximately 10^8^ CFU/mL, while viable *L. paracasei* numbers ranged from 10^3^ to 10^8^ CFU/mL. Firstly, the accuracy of the established PMA-qPCR method within these range was validated. The accuracy profile facilitates the assessment of both accuracy and precision by comparing the measured values with their corresponding theoretical values (ISO 16140-2, 2016). Typically, an acceptability limit (AL) of ±0.5 Log_10_ units is employed to delineate the permissible difference between the measured and theoretical values. This AL represents the maximum allowable deviation of the method from the theoretical values.

**Figure 5:**
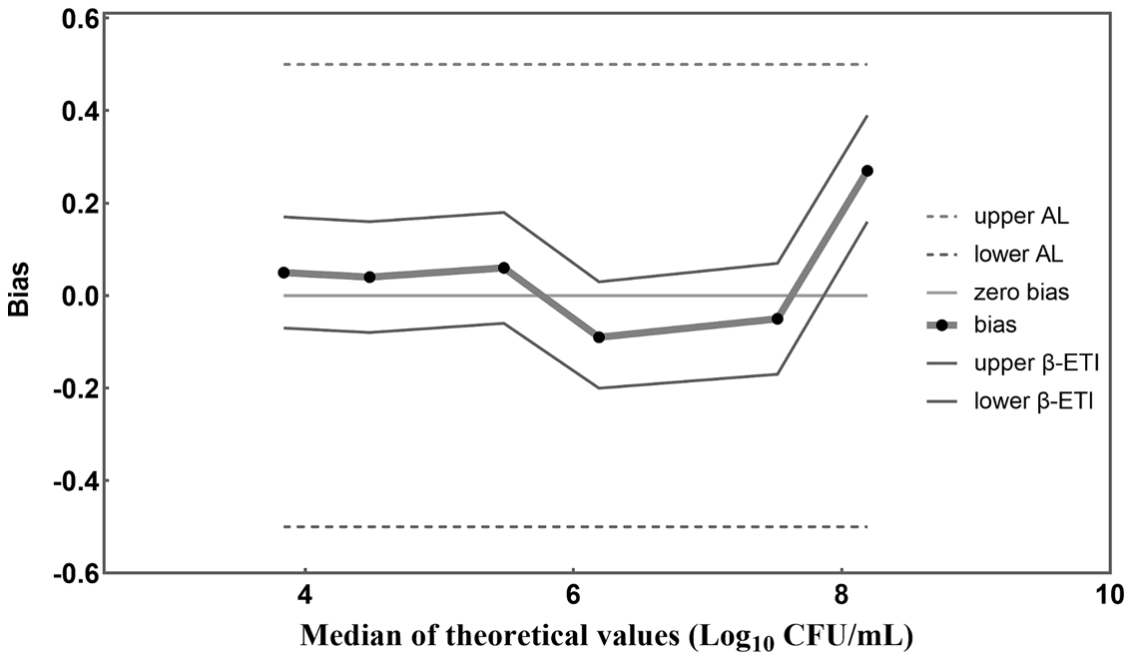
Accuracy profile for different concentrations of *L. paracasei* detected by the established PMA-qPCR method. The β-ETI represents the interval within which the expected proportion of future results will fall, with β set at 80% in accordance with ISO 16140-2:2016 (E) for this study. The bias (Bi) was determined as the absolute difference between the medians of the theoretical and measured values.

The results obtained from PMA-qPCR detection were statistically analyzed according to ISO 16140-2 (2016) (*E*). A graphical representation of computed results was created, with the horizontal axis depicting theoretical values in Log_10_ units and the vertical axis illustrating the bias (Figure 5). Straight lines connect the upper and lower tolerance-interval limits to interpolate the behavior of the limits across different levels of the validation samples. The horizontal line denotes the theoretical values, while any disparities between theoretical values and average concentration levels of *L. paracasei* are depicted by black dots. In the absence of biases, these recovered values align with the horizontal theoretical line. Additionally, AL is indicated by two dashed horizontal lines, and β-ETI (expected tolerance interval) limits are shown as broken full lines. According to Figure 5, the bias between theoretical and measured values for each viable cell concentration was 0.05, 0.04, 0.06, −0.09, −0.05, and 0.27 Log_10_ units. Importantly, all these biases are all within the acceptable limits (± 0.5 Log_10_ units). This demonstrated the compelling evidence for the accuracy of the PMA-qPCR method in quantifying viable *L. paracasei* within 10^3^–10^8^ CFU/mL. Furthermore, five replicates were conducted for each sample to evaluate the precision of the established PMA-qPCR method. The coefficient of variation (CV) for the Log_10_-transformed viable cell counts were calculated. Low CV values of 0.97, 0.39, 1.66, 1.61, 2.11, and 1.74%, underscored the robustness of the PMA-qPCR method in accurately quantifying viable cell counts.

**Figure 6:**
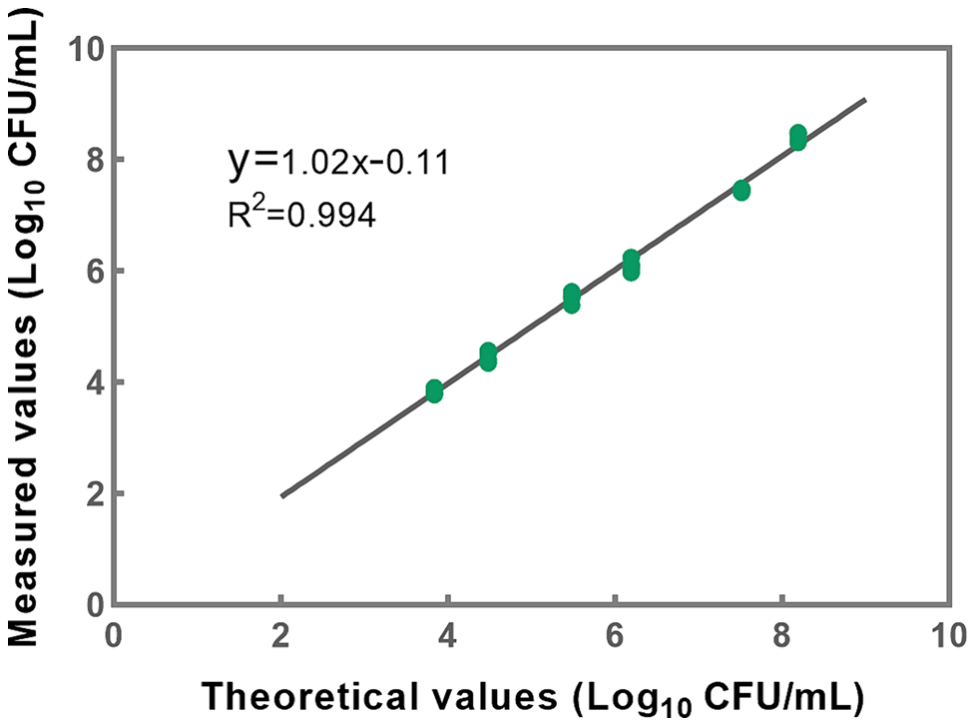
A linear correlation between theoretical and measured values was effectively established within the concentration range of 10^3^ to 10^8^ CFU/mL for *L. paracasei*. Each data point presented herein is derived from the analysis of five replicates.

Based on the accuracy results, a linear regression analysis was conducted to correlate the theoretical values with the measured values (Figure 6). The resulting correlation coefficient (R^2^) of the fitted curve was determined to be 0.994, indicating a strong linear relationship between the measured and theoretical values within the range of 10^3^ to 10^8^ CFU/mL (Figure 6). These findings substantiate the method’s reliability and accuracy in quantifying bacterial concentrations within the specified range. However, it is noteworthy that the upper limit of the quantitative range is set at 10^8^ CFU/mL, reflecting the limit of detection rather than necessarily delineating the genuine upper threshold of the developed methodology. In cases where bacterial densities surpass this concentration, the total bacterial density can be adjusted to 10^8^ CFU/mL, following which the optimal conditions for PMA treatment can be applied to the sample.

### 3.6 Applications of PMA-qPCR method to different sample types

**Figure 7:**
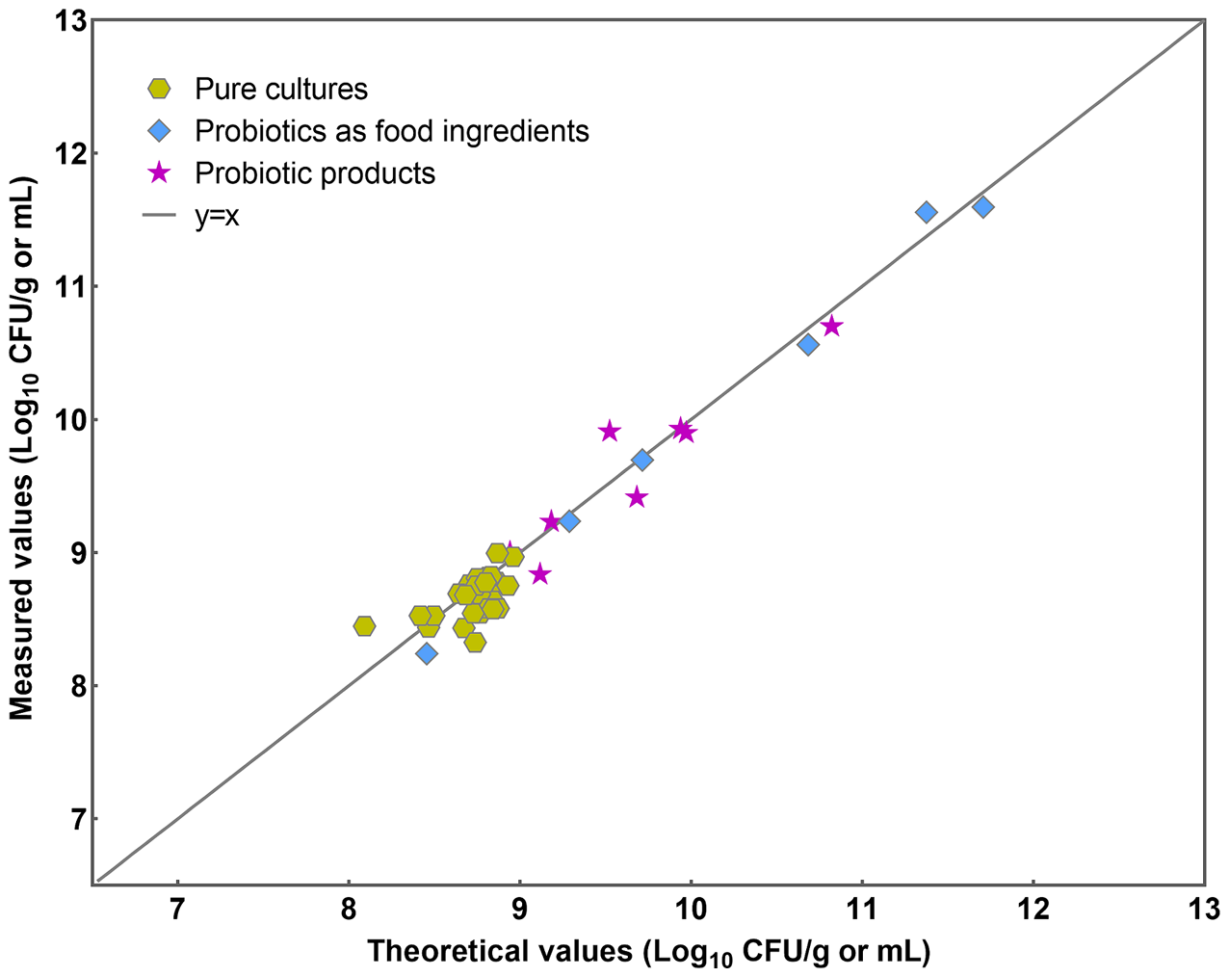
Scatter plot of theoretical values versus measured results.

The implemented PMA-qPCR method was employed across three distinct sample categories: pure cultures, probiotics as food constituents, and probiotic products. Pure cultures denote samples exclusively containing bacteria devoid of any matrix influence. Probiotic as food ingredients encompass samples containing either single strains of *L. paracasei* or multiple bacterial strains, with little matrix influence. Probiotic products encompass samples containing multiple bacterial strains along with complex matrix effects. Initially, a y = x line was plotted to visualize the level of agreement between the theoretical and measured values (Figure 7). Most data points closely conformed to the line for each analyzed sample, indicating a high level of concordance between the theoretical and measured values.

**Figure 8:**
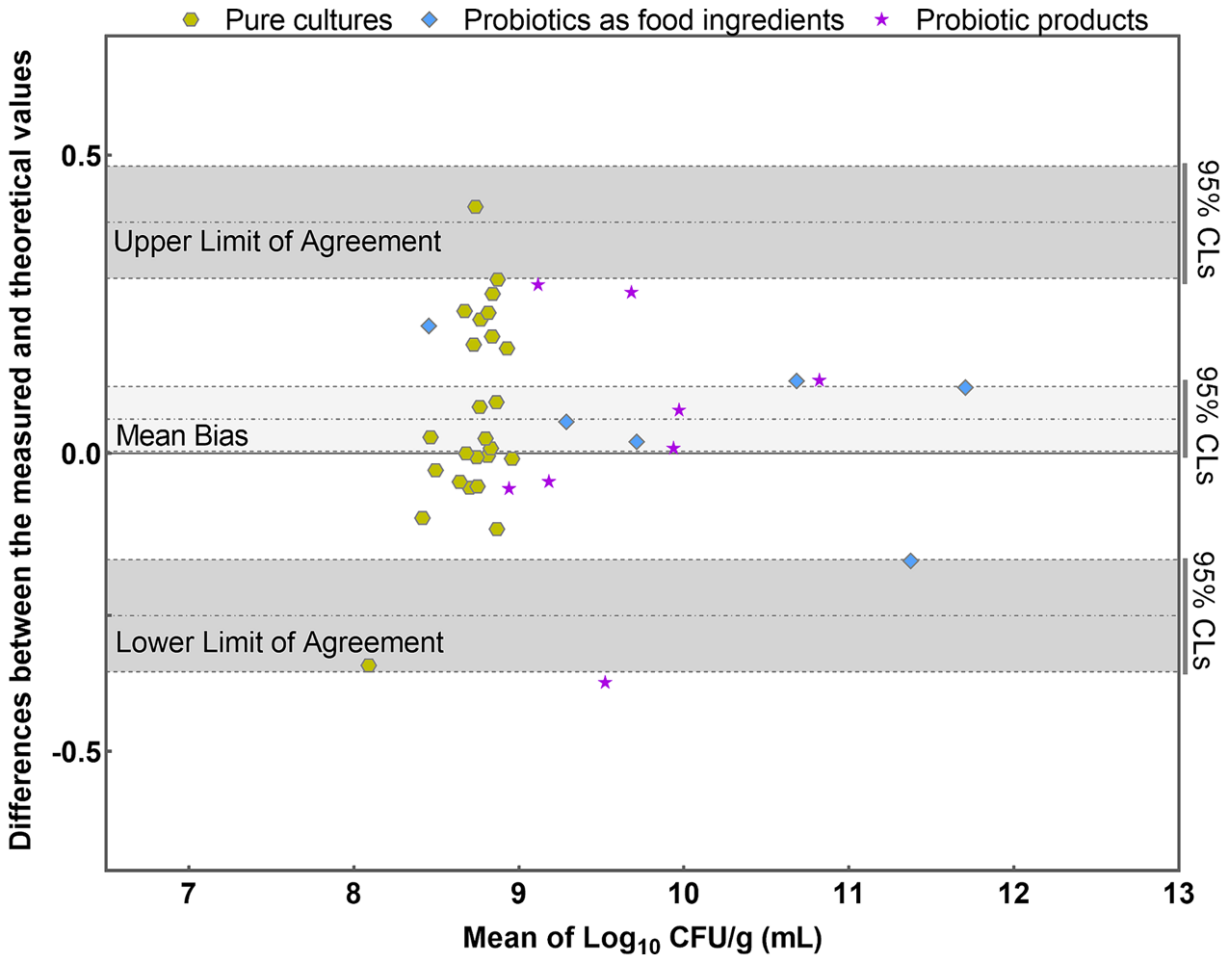
Bland–Altman plots comparing the quantitative values of PMA-qPCR measured values with those of theoretical values for 39 samples. (mean difference = 0.058 Log_10_; SD = 0.168 Log_10_).

The results were further analyzed using the Bland–Altman method in accordance with ISO 16140-2 (2016) (*E*). Individual sample differences against the mean values were plotted, showing the line of identity (zero difference), the line of bias, and the upper and lower 95% confidence limits (CLs) of agreement for the bias (Figure 8). The mean bias of the 39 samples was 0.058 Log_10_, demonstrating high agreement between PMA-qPCR measured and theoretical values. The lower and upper limits of agreement were − 0.272 and 0.388 Log_10_. The 95% confidence limits were − 0.366 to −0.178 Log_10_ and 0.294 to 0.482 Log_10_, respectively (Figure 8). The differences between the measured and theoretical values of 38 samples consistently fell within the 95% confidence interval defined by the CLs. Only one probiotic sample exceeded the CLs, aligning with ISO 16140, which allows no more than 1 out of 20 data points to exceed the CLs. For this outlying sample, the difference between the measured and theoretical values was −0.385 Log_10_, still within ±0.5 Log_10_. This demonstrates the accuracy and suitability of the PMA-qPCR method for quantifying viable *L. paracasei*. A *T*-test was utilized to assess the significance of differences between the theoretical and measured values of all 39 samples. The resulting *p* value of 0.76 (*p* > 0.05) indicates no significant difference between the theoretical and measured groups within the sample set. This underscores the precision and reliability of the PMA-qPCR method for detecting viable cells across various applications, including pure cultures, probiotics as food ingredients, and composite probiotic products.

**Figure 9:**
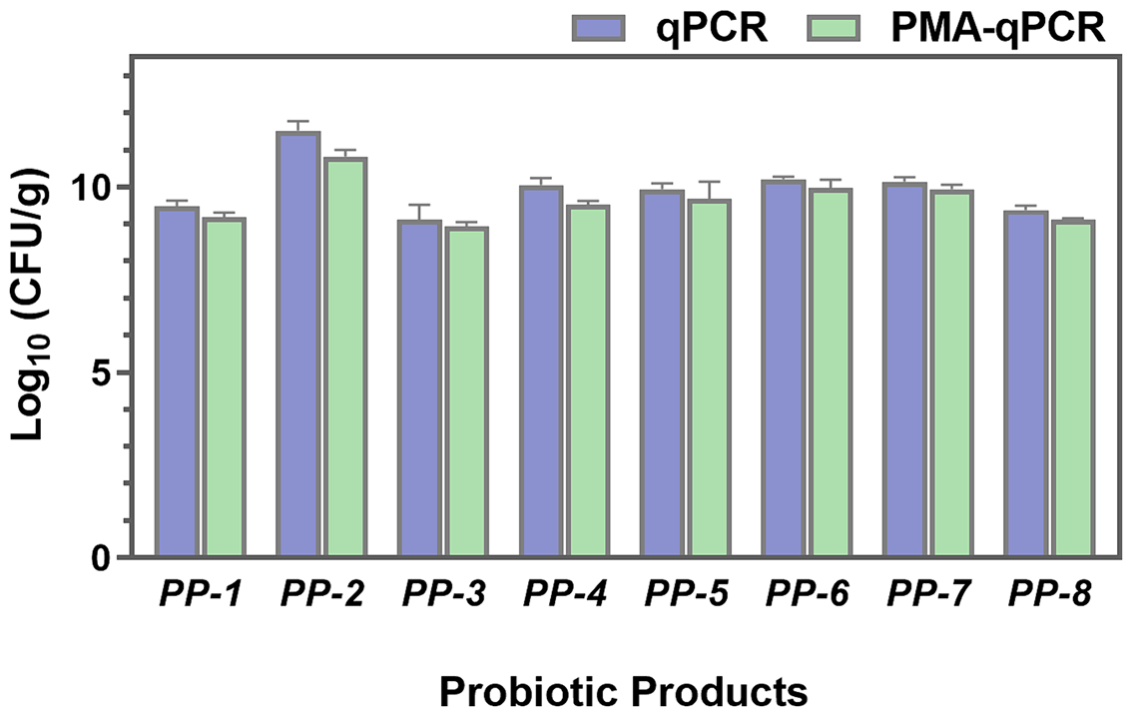
The total cell numbers (qPCR) and viable cell numbers (PMA-qPCR) of *L. paracasei* in eight probiotic products (PP-1 to PP-8) were assessed.

To investigate the total, viable, and dead cells in probiotic products, both PMA-treated and untreated samples were analyzed using qPCR. As shown in Figure 9, the total cell numbers (qPCR) of *L. paracasei* were higher than the viable cell numbers (PMA-qPCR). Significant differences were particularly observed in PP-2 and PP-4 probiotic products, with *p* values lower than 0.05. These findings indicate the presence of dead or membrane-damaged cells in the probiotic products. The PMA-qPCR method effectively excluded dead or membrane-damaged cells, providing an accurate count of viable *L. paracasei* cells in composite probiotics.

## 4 Discussion

The specificity of the newly designed primer for *L. paracasei* is paramount for ensuring precise detection and quantification of this probiotic strain, particularly within complex sample matrices (Broeders et al., 2014; Garrido-Maestu et al., 2018), significantly enhancing probiotic product manufacturing and quality control. Our primer design methodology is founded on a meticulous analysis of genomic data, leveraging 176 publicly available *L. paracasei* genomes subjected to rigorous re-annotation to ensure data consistency. This comprehensive approach facilitated an accurate gene presence/absence analysis, crucial for identifying core genes specific to *L. paracasei*, ensuring the target sequence is present across all strains while absent in non-target organisms. Utilizing CD-HIT software for gene presence/absence analysis at the protein level enabled precise clustering of similar proteins, identifying conserved gene families with high confidence. Stringent thresholds (50% pairwise identity and 0.7 length difference) ensured the inclusion of genuinely conserved genes, enhancing the specificity of the target. Following identification of potential core genes, their nucleotide sequences underwent BLASTN analysis against the NCBI Nucleotide collection to validate their specificity to *L. paracasei* at the nucleotide level, eliminating significant similarities with non-target species. This dual-level verification, examining both protein and nucleotide levels, provided a robust foundation for designing highly specific primers. The selection of the gene encoding Alkaline Shock Protein 23 was based on its consistent presence across *L. paracasei* strains and absence in related species, with its stability and crucial role in stress response mechanisms contributing to its conservation as an ideal marker for species-specific detection. Validation processes further reinforced the primer’s specificity, with *in silico* tests using Primer-BLAST against extensive whole-genome sequences from target and non-target strains confirming the absence of significant similarity with non-target organisms. Practical inclusivity and exclusivity tests involving 25 *L. paracasei* strains and 37 non-target strains provided empirical evidence of the primer’s accurate discrimination between target and non-target DNA (Figure 2), underscoring the primer’s reliability and specificity for diverse applications in probiotic research and product development.

The efficiency of DNA extraction is pivotal for accurately quantifying target microorganisms, with minimizing DNA loss being a crucial aspect in maintaining precision. Although commercial DNA extraction kits are widely used, concerns regarding DNA loss during column purification have been frequently documented, often attributed to the competitive binding of humic substances to silica membranes (Lloyd et al., 2010; Natarajan et al., 2016; Plotka et al., 2017). In this study, a streamlined approach to DNA extraction was employed, utilizing a one-step cell lysis method with a bead mill homogenizer due to its rapidity and ease of operation. This simplification of procedures significantly reduces the potential for DNA loss, thereby enhancing the reliability of downstream analyses. Moreover, maintaining consistent lysis conditions, including speed and duration, is essential for ensuring the stability and reproducibility of DNA quality. Effective DNA extraction is paramount as it lays the groundwork for establishing a robust correlation (*R*^2^ = 0.997) between C_q_ values and viable cell numbers, as depicted in Figure 4. Consequently, the resulting standard curve facilitates the translation of DNA quantities into viable cell numbers (Ilha et al., 2016; Yang et al., 2021). Furthermore, the efficacy of this method extends across various sample types, including pure cultures, probiotics as food ingredients, and compound probiotic products, as evidenced by the high degree of consistency between theoretical and PMA-qPCR measured values of viable *L. paracasei* (Figures 7, 8). Previous research has also demonstrated the accuracy of the bead-beating method in quantifying viable *L. rhamnosus* cell numbers (Guo et al., 2024), further affirming the advantages of this DNA extraction approach. Therefore, the bead-beating method emerges as a highly recommended tool for obtaining DNA followed by qPCR amplification, facilitating accurate viable cell counts and enhancing the reliability of microbial analysis in diverse sample matrices.

The PMA treatment condition is a crucial parameter for accurate viable cell counting, as it directly impacts the efficiency of dead cell or extracellular DNA filtration while leaving live cells unaffected (Nocker et al., 2006; Fujimoto and Watanabe, 2013; Yang et al., 2021). In this study, the chosen PMA treatment conditions (50 μM/L, 5 min, 15 min) (Desfossés-Foucault et al., 2012; Villarreal et al., 2013) were validated as optimal for distinguishing between viable and dead cells under bacterial concentrations of 10^8^ CFU/mL (Figure 3). A notable aspect of this study is the comprehensive collection of 25 distinct strains of *L. paracasei* to confirm the PMA treatment conditions, which is rare in previous PMA-qPCR studies. The results presented in Figure 3 demonstrate the wide applicability of the optimal PMA treatment conditions across various strain types, including both laboratory strains and commercial ones. This highlights the robustness and versatility of the selected PMA treatment protocol for accurately distinguishing viable cells from dead ones, regardless of strain origin or source.

The developed PMA-qPCR method demonstrates high accuracy in quantifying viable *L. paracasei* across a broad range of concentrations and sample types. The strong correlation coefficient (*R*^2^ = 0.994) observed in the linear analysis within the concentration range of 10^3^ to 10^8^ CFU/mL (Figure 6) underscores the method’s reliability in quantifying viable cell numbers. This high degree of linearity indicates the precise ability of the PMA-qPCR method to maintain accuracy and consistency across varying levels of viable cells. Furthermore, the established PMA-qPCR method underwent validation across three dimensions of sample types: pure cultures, probiotics as food ingredients, and compound probiotic products (Figures 7, 8). For instance, in compound probiotic products containing multiple bacterial strains, such as *B. animals* subsp. *lactis*, *L. rhamnosus*, *L. paracasei*, *L. plantarum*, *L. fermentum*, *L. gasseri*, *B. breve*, *L. delbrueckii* subsp. *bulgaricus*, *L. reuteri* and others, the difference between theoretical and PMA-qPCR measured values was −0.059 Log_10_ (Figure 8), demonstrating the high accuracy of the method in quantifying viable *L. paracasei* within complex probiotics. The accurate results obtained in this study further validate the specificity of the primers, affirming their capability to detect *L. paracasei* without interference from other non-target bacteria. Additionally, the compound probiotic products contained various matrix components such as resistant dextrin, erythritol, maltitol, polydextrose, or fructooligosaccharides. The successful quantification of viable *L. paracasei* within these complex matrices highlights the robustness and tolerance of the PMA treatment conditions and qPCR reaction to diverse sample compositions. Although utilizing the DNA of the target strain for preparing the standard curve theoretically enhances accuracy by tailoring the qPCR amplification process (Ilha et al., 2016; Odooli et al., 2018; Scariot et al., 2018), practical scenarios often involve unknown or unobtainable target bacterial strains. The high accuracy demonstrated in this study suggests the feasibility of applying a standard curve derived from the type strain to other strains within the same species. The comprehensive validation process and results provide a thorough overview confirming the suitability of the key parameters chosen, including DNA extraction, PMA treatment conditions, and standard curve preparation, for establishing the PMA-qPCR method. This underscores the method’s versatility and suitability for assessing bacterial viability in real-world samples with diverse compositions.

This study further demonstrated the presence of dead/damaged cells in probiotic products, as illustrated in Figure 9. During production, storage, and distribution, probiotic products are subjected to various biological, physical, and chemical stresses. These stresses can damage the probiotic cells, resulting in a microbial population comprising viable, dead, and stressed/damaged cells, including those in a viable but non-culturable (VBNC) state (Fiore et al., 2020; Fusco et al., 2021). The presence of dead and VBNC cells may impact the quality and efficacy of probiotic products (Foglia et al., 2020; Fusco et al., 2021). Therefore, accurate quantification of viable cells in probiotic products is crucial to ensure their effectiveness. The PMA-qPCR method represents a significant advancement in the accurate identification of probiotics and the quantification of viable bacteria. Both the findings of this study and previous research endeavors have unequivocally demonstrated the method’s accuracy and stability in achieving precise identification of target strains and enumeration of viable bacteria (Berezhnaya et al., 2021; Yang et al., 2021; Guo et al., 2024). Furthermore, the method’s versatility allows for the simultaneous detection of multiple bacteria under the same PMA-qPCR conditions, thereby enhancing efficiency. This capability holds profound implications for consistency control in enterprise production processes and market supervision of compound probiotics. The simplicity and rapidity of the PMA-qPCR method make it highly conducive to standardized research and application. Its feasibility in routine use provides invaluable technical support for ensuring the quality and safety of probiotic products. By offering a reliable means of quantifying viable bacteria, the method contributes to enhancing the transparency and accountability of probiotic product labeling, thereby bolstering consumer confidence.

## 5 Conclusion

In conclusion, this study has successfully developed and validated a precise PMA-qPCR method for quantifying viable *L. paracasei* in probiotics. The specificity of the newly designed primers was rigorously evaluated, demonstrating high specificity for *L. paracasei* detection across various strains. Optimal PMA treatment conditions were established to effectively distinguish between viable and dead cells, ensuring accurate quantification of viable *L. paracasei*. The method exhibited a strong linear relationship between C_q_ values and viable cell numbers, with high amplification efficiency and a quantification limit of 7.30 × 10^3^ CFU/mL. The accuracy and precision of the method were confirmed, with biases within acceptable limits across various concentrations of viable cells. Moreover, the method demonstrated robustness and reliability across different sample types, including pure cultures, probiotics as food ingredients, and compound probiotic products. Its simplicity, speed, and consistency make it indispensable for standardized research, offering vital technical support for quality assurance in probiotic product manufacturing. Its implementation in routine testing procedures can enhance transparency and accountability in the probiotics industry, ultimately bolstering consumer confidence and satisfaction.

## Data availability statement

The original contributions presented in the study are included in the article/Supplementary material, further inquiries can be directed to the corresponding author/s.

## Author contributions

LG: Visualization, Writing – review & editing, Writing – original draft, Validation, Methodology, Data curation. XZe: Writing – original draft, Writing – review & editing, Project administration, Methodology, Funding acquisition. YJia: Writing – review & editing, Writing – original draft, Methodology. CS: Writing – review & editing, Methodology, Data curation. XZh: Writing – review & editing, Data curation. ZS: Writing – review & editing, Software, Methodology, Data curation. SM: Visualization, Writing – review & editing, Software, Methodology. YL: Writing – review & editing, Project administration, Methodology. YG: Writing – review & editing, Project administration, Methodology. YJin: Writing – review & editing, Methodology. SY: Writing – review & editing, Supervision, Project administration, Funding acquisition.

## Footnotes

^1^https://www.ncbi.nlm.nih.gov/tools/primer-blast/index.cgi?LINK_LOC=BlastHome

^2^https://www.ncbi.nlm.nih.gov/genome/

## References

[ref1] Altschul S. F. Gish W. Miller W. Myers E. W. Lipman D. J. (1990). Basic local alignment search tool. J. Mol. Biol. 215, 403–410. doi: 10.1016/S0022-2836(05)80360-2 2231712

[ref2] Beck L. C. Masi A. C. Young G. R. Vatanen T. Lamb C. A. Smith R.. (2022). Strain-specific impacts of probiotics are a significant driver of gut microbiome development in very preterm infants. Nat. Microbiol. 7, 1525–1535. doi: 10.1038/s41564-022-01213-w, PMID: 36163498 PMC9519454

[ref3] Berezhnaya Y. Bikaeva I. Gachkovskaia A. Demidenko A. Klimenko N. Tyakht A.. (2021). Temporal dynamics of probiotic Lacticaseibacillus casei and rhamnosus abundance in a fermented dairy product evaluated using a combination of cultivation-dependent and -independent methods. LWT 148:111750. doi: 10.1016/j.lwt.2021.111750

[ref4] Beverage Y. Vitheejongjaroen P. Phettakhu P. Arsayot W. Taweechotipatr M. (2024). The ability of Lacticaseibacillus paracasei MSMC 36–9 strain with probiotic potential to ferment coconut Milk and produce a Yogurt-Type Beverage. Beverages 10:30. doi: 10.3390/beverages10020030

[ref5] Boyte M. E. Benkowski A. Pane M. Shehata H. R. (2023). Probiotic and postbiotic analytical methods: a perspective of available enumeration techniques. Front. Microbiol. 14, 1–15. doi: 10.3389/fmicb.2023.1304621, PMID: 38192285 PMC10773886

[ref6] Broeders S. Huber I. Grohmann L. Berben G. Taverniers I. Mazzara M.. (2014). Guidelines for validation of qualitative real-time PCR methods. Trends Food Sci. Technol. 37, 115–126. doi: 10.1016/j.tifs.2014.03.008

[ref8] Chiang S. S. Pan T. M. (2012). Beneficial effects of *Lactobacillus paracasei* subsp. paracasei NTU 101 and its fermented products. Appl. Microbiol. Biotechnol. 93, 903–916. doi: 10.1007/s00253-011-3753-x 22159887

[ref10] Chuang L. C. Huang C. S. Ou-Yang L. W. Lin S. Y. (2011). Probiotic *Lactobacillus paracasei* effect on cariogenic bacterial flora. Clin. Oral Investig. 15, 471–476. doi: 10.1007/s00784-010-0423-9, PMID: 20502929 PMC3133768

[ref11] Congjie H. Cui C. Cai Y. Zhou Y. Wang J. (2024). Strain-specific benefits of bacillus probiotics in hybrid grouper: growth enhancement, metabolic health, immune modulation, and vibrio harveyi resistance. Animals 7:1062. doi: 10.3390/ani14071062 PMC11011011 38612301

[ref12] Desfossés-Foucault É. Dussault-Lepage V. Le Boucher C. Savard P. LaPointe G. Roy D. (2012). Assessment of probiotic viability during Cheddar cheese manufacture and ripening using propidium monoazide-PCR quantification. Front. Microbiol. 3, 1–11. doi: 10.3389/fmicb.2012.00350, PMID: 23060868 PMC3463833

[ref13] Douglas C. A. Ivey K. L. Papanicolas L. E. Best K. P. Muhlhausler B. S. Rogers G. B. (2020). DNA extraction approaches substantially influence the assessment of the human breast milk microbiome. Sci. Rep. 10, 123–110. doi: 10.1038/s41598-019-55568-y, PMID: 31924794 PMC6954186

[ref14] Elsalam K. A. A. (2003). Bioinformatic tools and guideline for PCR primer design. Afr. J. Biotechnol. 2, 91–95. doi: 10.5897/AJB2003.000-1019

[ref15] Falfán-Cortés R. N. Mora-Peñaflor N. Gómez-Aldapa C. A. Rangel-Vargas E. Acevedo-Sandoval O. A. Franco-Fernández M. J.. (2022). Characterization and evaluation of the probiotic potential in vitro and in situ of Lacticaseibacillus paracasei isolated from Tenate cheese. J. Food Prot. 85, 112–121. doi: 10.4315/JFP-21-021, PMID: 34324685

[ref16] Fenster K. Freeburg B. Hollard C. Wong C. Laursen R. R. Ouwehand A. C. (2019). The production and delivery of probiotics: a review of a practical approach. Microorganisms 7, 1–17. doi: 10.3390/microorganisms7030083, PMID: 30884906 PMC6463069

[ref17] Fiore W. Arioli S. Guglielmetti S. (2020). The neglected microbial components of commercial probiotic formulations. Microorganisms 8:1177. doi: 10.3390/microorganisms8081177, PMID: 32756409 PMC7464440

[ref18] Foglia C. Allesina S. Amoruso A. De Prisco A. Pane M. (2020). New insights in enumeration methodologies of probiotic cells in finished products. J. Microbiol. Methods 175:105993. doi: 10.1016/j.mimet.2020.105993, PMID: 32621828

[ref19] Fujimoto S. Nakagami Y. Kojima F. (2004). Optimal bacterial DNA isolation method using bead-beating technique. Mem. Kyushu Univ Dep Health Scis Med. Sch. 3, 33–38.

[ref20] Fujimoto J. Watanabe K. (2013). Quantitative detection of viable *Bifidobacterium bifidum* BF-1 cells in human feces by using propidium monoazide and strain-specific primers. Appl. Environ. Microbiol. 79, 2182–2188. doi: 10.1128/AEM.03294-12, PMID: 23354719 PMC3623260

[ref21] Fusco V. Fanelli F. Chieffi D. (2021). Authenticity of probiotic foods and dietary supplements: a pivotal issue to address. Crit. Rev. Food Sci. Nutr. 62, 6854–6871. doi: 10.1080/10408398.2021.1907300, PMID: 33819118

[ref22] Garrido-Maestu A. Azinheiro S. Carvalho J. Prado M. (2018). Rapid and sensitive detection of viable *Listeria monocytogenes* in food products by a filtration-based protocol and qPCR. Food Microbiol. 73, 254–263. doi: 10.1016/j.fm.2018.02.004, PMID: 29526210

[ref23] Guo L. Wan K. Zhu J. Ye C. Chabi K. Yu X. (2020). Detection and distribution of VBNC/viable pathogenic Bacteria in full-scale drinking water treatment plants. J. Hazard. Mater. 406:124335. doi: 10.1016/j.jhazmat.2020.124335, PMID: 33160785

[ref24] Guo L. Ze X. Feng H. Liu Y. Ge Y. Zhao X.. (2024). Identification and quantification of viable Lacticaseibacillus rhamnosus in probiotics using validated PMA-qPCR method. Front. Microbiol. 15, 1–12. doi: 10.3389/fmicb.2024.1341884, PMID: 38298895 PMC10828034

[ref25] Hellebois T. Canuel R. Leclercq C. C. Gaiani C. Soukoulis C. (2024). Milk protein-based cryogel monoliths as novel encapsulants of probiotic bacteria. Part II: Lacticaseibacillus rhamnosus GG storage stability and bioactivity under in vitro digestion. Food Hydrocoll. 146:109173. doi: 10.1016/j.foodhyd.2023.109173

[ref26] Ilha E. C. Scariot M. C. Treml D. Pereira T. P. Sant Anna E. S. Prudêncio E. S.. (2016). Comparison of real-time PCR assay and plate count for *Lactobacillus paracasei* enumeration in yoghurt. Ann. Microbiol. 66, 597–606. doi: 10.1007/s13213-015-1137-7

[ref27] ISO 16140-2 (2016). ISO 16140-2, microbiology of the food chain — method validation — part 2: protocol for the validation of alternative (proprietary) methods against a reference method. Available at: https://www.iso.org/obp/ui/en/#iso:std:iso:16140:-2:ed-1:v1:en

[ref28] Jang H. J. Lee N. K. Paik H. D. (2019). Probiotic characterization of *Lactobacillus brevis* KU15153 showing antimicrobial and antioxidant effect isolated from kimchi. Food Sci. Biotechnol. 28, 1521–1528. doi: 10.1007/s10068-019-00576-x, PMID: 31695951 PMC6811472

[ref29] Kiousi D. E. Karadedos D. M. Sykoudi A. Repanas P. Kamarinou C. S. Argyri A. A.. (2023). Development of a multiplex PCR assay for efficient detection of two potential probiotic strains using whole genome-based primers. Microorganisms 11:2553. doi: 10.3390/microorganisms11102553, PMID: 37894211 PMC10609308

[ref30] Kumar H. Salminen S. Verhagen H. Rowland I. Heimbach J. Bañares S.. (2015). Novel probiotics and prebiotics: road to the market. Curr. Opin. Biotechnol. 32, 99–103. doi: 10.1016/j.copbio.2014.11.021, PMID: 25499742

[ref31] Kwon H. S. Yang E. H. Lee S. H. Yeon S. W. Kang B. H. Kim T. Y. (2005). Rapid identification of potentially probiotic Bifidobacterium species by multiplex PCR using species-specific primers based on the region extending from 16S rRNA through 23S rRNA. FEMS Microbiol. Lett. 250, 55–62. doi: 10.1016/j.femsle.2005.06.041, PMID: 16039804

[ref32] Latka A. Simaey L. Van Reynders M. Cools P. Rogier T. (2022). Optimization of propidium monoazide qPCR (Viability-qPCR) to quantify the killing by the Gardnerella-Specific Endolysin PM-477, directly in vaginal samples from women with bacterial vaginosis. Antibiotics 11:111. doi: 10.3390/antibiotics11010111 35052988 PMC8773202

[ref33] Lawley B. Munro K. Hughes A. Hodgkinson A. J. Prosser C. G. Lowry D.. (2017). Differentiation of *bifidobacterium longum* subspecies longum and infantis by quantitative PCR using functional gene targets. PeerJ 5, e3375–e3314. doi: 10.7717/peerj.3375, PMID: 28560114 PMC5446769

[ref34] Li W. Godzik A. (2006). Cd-hit: a fast program for clustering and comparing large sets of protein or nucleotide sequences. Bioinformatics 22, 1658–1659. doi: 10.1093/bioinformatics/btl158 16731699

[ref35] Li W. Wooley J. C. Godzik A. (2008). Probing metagenomics by rapid cluster analysis of very large datasets. PLoS One 3, 1–7. doi: 10.1371/journal.pone.0003375, PMID: 18846219 PMC2557142

[ref36] Lloyd K. G. MacGregor B. J. Teske A. (2010). Quantitative PCR methods for RNA and DNA in marine sediments: maximizing yield while overcoming inhibition. FEMS Microbiol. Ecol. 72, 143–151. doi: 10.1111/j.1574-6941.2009.00827.x, PMID: 20059545

[ref37] Marole T. A. Sibanda T. Buys E. M. (2024). Assessing probiotic viability in mixed species yogurt using a novel propidium monoazide (PMAxx)-quantitative PCR method. Front. Microbiol. 15:1325268. doi: 10.3389/fmicb.2024.1325268, PMID: 38389538 PMC10882272

[ref39] Natarajan V. P. Zhang X. Morono Y. Inagaki F. Wang F. (2016). A modified SDS-based DNA extraction method for high quality environmental DNA from seafloor environments. Front. Microbiol. 7:986. doi: 10.3389/fmicb.2016.00986, PMID: 27446026 PMC4917542

[ref40] Nocker A. Cheung C. Y. Camper A. K. (2006). Comparison of propidium monoazide with ethidium monoazide for differentiation of live vs. dead bacteria by selective removal of DNA from dead cells. J. Microbiol. Methods 67, 310–320. doi: 10.1016/j.mimet.2006.04.015, PMID: 16753236

[ref41] Odooli S. Khalvati B. Safari A. Mehraban M. H. Kargar M. Ghasemi Y. (2018). Comparison of tuf gene-based qPCR assay and selective plate count for *Bifidobacterium animalis* subsp. lactis BB-12 quantification in commercial probiotic yoghurts. Int. Food Res. J. 25, 1708–1719.

[ref42] Pérez Martínez G. Giner-Pérez L. Castillo-Romero K. F. (2023). Bacterial extracellular vesicles and associated functional proteins in fermented dairy products with *Lacticaseibacillus paracasei*. Front. Microbiol. 14:1165202. doi: 10.3389/fmicb.2023.1165202, PMID: 37152726 PMC10157241

[ref43] Plotka M. Wozniak M. Kaczorowski T. (2017). Quantification of plasmid copy number with single colour droplet digital PCR. PLoS One 12, 1–17. doi: 10.1371/journal.pone.0169846, PMID: 28085908 PMC5234771

[ref45] Principi N. Cozzali R. Farinelli E. Brusaferro A. Esposito S. (2018). Gut dysbiosis and irritable bowel syndrome: the potential role of probiotics. J. Infect. 76, 111–120. doi: 10.1016/j.jinf.2017.12.013 29291933

[ref46] Quin C. Estaki M. Vollman D. M. Barnett J. A. Gill S. K. Gibson D. L. (2018). Probiotic supplementation and associated infant gut microbiome and health: a cautionary retrospective clinical comparison. Sci. Rep. 8, 1–17. doi: 10.1038/s41598-018-26423-3, PMID: 29844409 PMC5974413

[ref47] Rogers-Broadway K. R. Karteris E. (2015). Amplification efficiency and thermal stability of qPCR instrumentation: current landscape and future perspectives. Exp. Ther. Med. 10, 1261–1264. doi: 10.3892/etm.2015.2712, PMID: 26622475 PMC4578049

[ref48] Ruijter J. M. Barnewall R. J. Marsh I. B. Szentirmay A. N. Quinn J. C. Van Houdt R.. (2021). Efficiency correction is required for accurate quantitative PCR analysis and reporting. Clin. Chem. 67, 829–842. doi: 10.1093/clinchem/hvab052, PMID: 33890632

[ref49] Ruijter J. M. Ramakers C. Hoogaars W. M. H. Karlen Y. Bakker O. van den Hoff M. J. B.. (2009). Amplification efficiency: linking baseline and bias in the analysis of quantitative PCR data. Nucleic Acids Res. 37:e45. doi: 10.1093/nar/gkp045, PMID: 19237396 PMC2665230

[ref50] Scariot M. C. Venturelli G. L. Prudêncio E. S. Arisi A. C. M. (2018). Quantification of *Lactobacillus paracasei* viable cells in probiotic yoghurt by propidium monoazide combined with quantitative PCR. Int. J. Food Microbiol. 264, 1–7. doi: 10.1016/j.ijfoodmicro.2017.10.021 29073460

[ref51] Shehata H. R. Hassane B. Newmaster S. G. (2023). Real-time polymerase chain reaction methods for strain specific identification and enumeration of strain Lacticaseibacillus paracasei 8700:2. Front. Microbiol. 13, 1–13. doi: 10.3389/fmicb.2022.1076631, PMID: 36741903 PMC9889646

[ref52] Shetty J. Mariyam D. (2020). The evolution of DNA extraction methods. Am. J. Biomed. Res. 8, 39–45. doi: 10.34297/ajbsr.2020.08.001234

[ref53] Sibanda T. Marole T. A. Thomashoff U. L. Thantsha M. S. Buys E. M. (2024). Bifidobacterium species viability in dairy-based probiotic foods: challenges and innovative approaches for accurate viability determination and monitoring of probiotic functionality. Front. Microbiol. 15, 1–21. doi: 10.3389/fmicb.2024.1327010, PMID: 38371928 PMC10869629

[ref54] Singh V. K. Mangalam A. K. Dwivedi S. Naik S. (1998). Primer premier: program for design of degenerate primers from a protein sequence. BioTechniques 24, 318–319. doi: 10.2144/98242pf02, PMID: 9494736

[ref55] Son S. H. Jeon H. L. Yang S. J. Sim M. H. Kim Y. J. Lee N. K.. (2018). Probiotic lactic acid bacteria isolated from traditional Korean fermented foods based on β-glucosidase activity. Food Sci. Biotechnol. 27, 123–129. doi: 10.1007/s10068-017-0212-1, PMID: 30263732 PMC6049735

[ref56] Song M. W. Kim K. T. Paik H. D. (2023). Probiotics as a functional health supplement in infant formulas for the improvement of intestinal microflora and immunity. Food Rev. Int. 39, 858–874. doi: 10.1080/87559129.2021.1928178

[ref57] Svec D. Tichopad A. Novosadova V. Pfaffl M. W. Kubista M. (2015). How good is a PCR efficiency estimate: recommendations for precise and robust qPCR efficiency assessments. Biomol. Detect. Quantif. 3, 9–16. doi: 10.1016/j.bdq.2015.01.005, PMID: 27077029 PMC4822216

[ref9001] The World Health Organization (2001). Health and nutritional properties of probiotics in food including powder Milk with live lactic acid Bacteria. FAO WHO. Available at: http://scholar.google.com/scholar?hl=en&btnG=Search&q=intitle:Health and Nutrit ional Properties of Probiotics in Food including Powder Milk with Live Lacti c Acid Bacteria#2%5Cnhttp://scholar.google.com/scholar?hl=en&btnG=Search&q=i ntitle:Health and Nutrit

[ref58] Villarreal M. L. Padilha M. Vieira A. D. Franco B. D. Martinez R. C. Saad S. M. (2013). Advantageous direct quantification of viable closely related probiotics in petit-suisse cheeses under in vitro gastrointestinal conditions by propidium monoazide - QPCR. PLoS One 8, 1–11. doi: 10.1371/journal.pone.0082102 PMC3866109 24358142

[ref59] Wang A. Zhong Q. (2024). Drying of probiotics to enhance the viability during preparation, storage, food application, and digestion: a review. Compr. Rev. Food Sci. Food Saf. 23, e13287–e13230. doi: 10.1111/1541-4337.13287, PMID: 38284583

[ref60] Yang Y. Liu Y. Shu Y. Xia W. Xu R. Chen Y. (2021). Modified PMA-qPCR method for rapid quantification of viable Lactobacillus spp. in fermented dairy products. Food Anal. Methods 14, 1908–1918. doi: 10.1007/s12161-021-02022-3

[ref61] Ye J. Coulouris G. Zaretskaya I. Cutcutache I. Rozen S. Madden T. L. (2012). Primer-BLAST: a tool to design target-specific primers for polymerase chain reaction. BMC Bioinformatics 13:134. doi: 10.1186/1471-2105-13-134 22708584 PMC3412702

[ref62] Zhang Y. Du R. Wang L. Zhang H. (2010). The antioxidative effects of probiotic *Lactobacillus casei* Zhang on the hyperlipidemic rats. Eur. Food Res. Technol. 231, 151–158. doi: 10.1007/s00217-010-1255-1

[ref63] Zhang S. J. Wang L. L. Lu S. Y. Hu P. Li Y. S. Zhang Y.. (2020). A novel, rapid, and simple PMA-qPCR method for detection and counting of viable Brucella organisms. J. Vet. Res. 64, 253–261. doi: 10.2478/jvetres-2020-0033, PMID: 32587912 PMC7305652

[ref64] Zhao L. Zhang D. Liu Y. Zhang Y. N. Meng D. Q. Xu Q.. (2022). Quantitative PCR assays for the strain-specific identification and enumeration of probiotic strain Lacticaseibacillus rhamnosus X253. Food 11:2282. doi: 10.3390/foods11152282 PMC9367767 35954048

